# Saccharin

**Published:** 1889-02-16

**Authors:** 


### SACCHARIN.

"Saccharin: Its Place in Pharmacy, with Formulæ," is the title of a pamphlet by Professor Attfield, F.R.S. The Professor states that saccharin will be of good service in pharmacy in four ways. First, it gives patients an opportunity of taking medicines in smaller bulk, a very minute proportion of saccharin being equal to a large bulk of sugar as regards sweetness; secondly, the intensity of the sweetness masks the taste of nauseous medicines; thirdly, patients who are obliged to avoid sugar may take saccharin; fourthly, saccharin does not ferment like sugar. Saccharin, it may be mentioned, is a substance comparatively recently brought into use, and obtained from the destructive distillation of coal-tar. And as is often the case with newly-discovered chemical preparations, saccharin was brought forward as useful in many ways and in various maladies. And, as also is just as often the case, it was not long before the utility of saccharin was denied. Especially was the utility of saccharin challenged by Continental physicians. Very recently, however, a "Vindication of Saccharin" has appeared in the shape of a pamphlet, published by Wilson, Salaman, and Co. There is a consensus of opinion that saccharin is a perfectly wholesome preparation, and may be used for purposes of sweetening in all cases where sugar is injurious, as it does not interfere with or impede the digestive process when taken in any practicable quantity.

